# Modern Sociology

**Published:** 1898-12-31

**Authors:** 


### Modern Sociology.

### THE LIQUOR TRADE IN AMERICA.

### III.—A Short Way With Drunkards.

No one wants a drunkard—no one, not even a publican. Naturally, employers of labour dislike a drunkard, as being an unreliable man, and for their own sakes they will try to check his excesses, even if they take no personal interest in the welfare of the man himself. The American Government report gives us some interesting information as to the efforts made by employers and the result attained.

We have before us the report of 3,726 establishments which admit that they have been troubled by drunkenness among their workers and have tried various means to lessen it. The means tried to check excess are numerous, more than 200 in all, but among all these some half-dozen stand out as the most general. These are discharge, warning, suspension, holding back wages, change of pay-day from Saturday, change of pay-day to Saturday, less frequent payment of wages, moral suasion, forbidding liquor to be brought on the premises, opposing the location of saloons near the premises, lessening the number of these or getting them closed. The table of results is not as large as that which tells us of the means employed. The large majority—2,887—of the firms which admit that they do try to keep their employés from drinking say nothing as to the outcome of their efforts, which we must therefore assume to be negative. Of those which tell us their results, the majority seem to find that discharge is the most effective plan. We presume that they speak from the point of view of their own interests rather than that of the worker, for while discharge from his employment may have a salutary effect on the man who is not yet a confirmed drunkard, by showing him the danger into which he is drifting, it is doubtful if that alone will do much to reform the really habitual drunkard; but 112 firms say that this method is successful, while only four say that they find it ineffective. Moral suasion, suspension, and warning seem to be regarded as effective by those who employ them, but they are unfortunately few in number, some 24 establishments in all. More interesting is the attempt to check drunkenness by changing the pay day from Saturday to some other day. The reason for this is clear enough. Saturday is the end of the week's work, and many men who, with a working day before them, would take care not to incapacitate themselves for it, allow themselves more licence on Saturday nights, with the prospect of the Sunday's rest. When they receive their pay, and have their money in hand, they are naturally inclined to spend it; but when they do not get it on Saturday fewer of them will risk spending it on drink. The good results claimed from the less frequent payment of wages belong to the same category. The assumption is that when the workman receives his wages he will go on a carouse, and the effort is merely to diminish his occasions for drinking. But we find that some establishments have actually changed their pay-day to Saturday. Why is this? The reason given is a sad one enough. It is that as the men will in any case get drunk on their pay-day, it is for the firm's benefit to pay them on Saturday, so that they may have the Sunday's rest in which to recover from their excesses. This method shows, perhaps, practical sense on the part of the employer, but hardly a high ideal of moral duty towards the employed.

One method requires special mention, though the results recorded from it are few—the preventing of the establishment of saloons, *i.e.*, public-houses, near the workshops. One firm says that this has been of use in diminishing drunkenness, and one says it has not, while 25 say that they have tried this, but do not tell with what results. At best, therefore, we cannot regard the success of this method as striking. So far as recorded, it has succeeded in one case and failed in another. We commend this fact to those enthusiasts who think that drunkenness can be largely diminished by the mere diminution of opportunities of drinking. In face of this, it is strange to find that the majority of the employers canvassed seemed to believe that prohibition will largely help to solve the drink problem. Of 4,914 who give suggestions as to the best methods of lessening the consumption of alcohol among the people, 1,103 recommend prohibition, and no other recommendation is nearly so well supported. Next in favour comes the recommendation not to employ drinking men, with 769 firms to back it; while in a descending scale we have high licence, education, the improvement of social conditions, Government control, and local option. As opposed to prohibition, we find some employers who would abolish all restrictions, and others who would fight the consumption of ardent spirits by encouraging the use of light wines and beer. It is to be feared that the two last recommendations will hardly gain in weight through the fact that they express the opinions of many liquor sellers. These gentlemen are all opposed to prohibition, which, they say, merely increases the desire for drink. And, indeed, we may so far agree with them as to admit that secret drinking is of all kinds the worst. But, on the other hand, they condemn the habit of "treating" as tending to lead to drunkenness, and this, it is true, often leads a man who would be content with one glass to take a great many.

Although the advice comes from a publican, we cannot but think that there is sound sense in the following dictum. The writer would conquer drunkenness "by having only pure liquors sold. The National or State Government should have liquors examined, and those not up to standard destroyed, as in the case of meat, milk, &c. The National Government should forbid the manufacture of continuous distillation (or quickaging goods, as they are sometimes called). They are ruinous to health. It should also set a period after which no spirituous liquors could be sold less than five years old." There can be no doubt that this advice, if it were followed, would have a considerable effect, not perhaps on drinking, but on drunkenness. The liquor sold to the working-class is often fearfully bad. It is coarse crude, fiery. The effect of it is to madden whoever consumes it, and leads to violence and often to crime, where even as large a quantity of better liquor would do them little if any harm, and would certainly not lead them into such dangerous excesses. "Whisky is a very bad thing," as the temperance reformer said. "Yes, especially bad whisky," as the experienced Highlander replied.

